# Endoscopy-Assisted Laparoscopic versus Laparoscopic Surgery for Gastrointestinal Stromal Tumor and the Impact on Patients' Coagulation, Surgical Condition, and Complications

**DOI:** 10.1155/2022/6847321

**Published:** 2022-03-23

**Authors:** Licheng Liu, Anna Dai

**Affiliations:** ^1^General Surgery Department (Two), Cangzhou Central Hospital, Cangzhou, Hebei, China; ^2^Meinian Health Clinic, Cangzhou, Hebei, China

## Abstract

**Objective:**

To assess the clinical efficiency of endoscopy-assisted laparoscopic versus laparoscopic surgery for gastrointestinal stromal tumors and the impact on patients' coagulation, surgical condition, and complications.

**Methods:**

Between November 2016 and May 2020, 126 eligible patients diagnosed with gastrointestinal stromal tumor (GIST) in our institution were recruited. They were concurrently randomly assigned at a ratio of 1 : 1 to receive either laparoscopic gastrectomy (reference group) or endoscopy-assisted laparoscopic gastrectomy (research group). The two groups were compared in terms of patients' coagulation function, surgical conditions, and complications.

**Results:**

The two groups had similar preoperative coagulation indices and the postoperative levels of activated partial thromboplastin time (APTT) and thromboplastin time (TT) (*P* > 0.05). Compared with the reference group, the research group showed lower PT levels (10.48 ± 0.68 vs. 11.97 ± 0.46) and higher FIB levels (0.67 ± 0.11 vs. 0.29 ± 0.07) (*P* < 0.05). Compared with the reference group, the study group had shorter operative time (81.21 ± 10.24 min versus 98.98 ± 15.31 min), shorter surgical incision (3.63 ± 1.12 cm versus 5.01 ± 1.14 cm), and less intraoperative bleeding (18.74 ± 6.98 ml versus 58.69 ± 15.87 ml) (*P* < 0.05). A markedly shorter length of hospital stay, time to the first postoperative exhaustion, and duration of drainage tube and gastric tube dwelling were observed in the research group versus the reference group (*P* < 0.05). The study group presented higher nutritional levels of patients at 3 days after surgery and a lower incidence of complication.

**Conclusion:**

Endoscopy-assisted laparoscopic treatment shows significant improvements in the efficiency of minimally invasive surgery and ensures a better prognosis and quality of life of patients with a good safety profile, so it is worthy of clinical application.

## 1. Introduction

Gastrointestinal stromal tumor (GIST) is a common malignancy that originates from the mesenchymal tissue of the gastrointestinal tract and accounts for most of the mesenchymal tumors in the gastrointestinal tract, with a high prevalence. The absence of specific clinical symptoms and the relatively insidious site of the lesion prevent effective diagnosis of the patient in the early stage [1, 2]. With the advancement of medical technology [3], laparoscopic gastrectomy is considered the mainstay for the treatment of GIST. It is a newly developed minimally invasive method with benefits such as small incisions, minimal scarring, reduced pain, fewer postoperative adhesions, and shorter hospital stays [4]. However, disadvantages such as incomplete surgical field of view and the complicated tumor resection due to the small size or insidiousness of tumors compromise the success of laparoscopic surgery, which desires improvements in clinical efficiency and safety [5, 6]. Endoscopy is a sophisticated imaging technology that provides a pictorial accuracy to allow the diagnosis of tumors with extremely small size [7]. Endoscopy-assisted laparoscopy avoids surgical blind spots to a greater extent to achieve better efficacy, taking into account the tumor size, location, and underlying disease in treatment regimen designs [8]. Previous research has reported significant enrichment in patients' postoperative recovery of endoscopy-assisted laparoscopic gastrectomy for GIST [9]. Here, endoscopy-assisted laparoscopic gastrectomy was adopted for the treatment of GIST resection, to provide a safer and more efficient treatment protocol. The results are as follows.

## 2. Materials and Methods

### 2.1. Baseline Data

**Figure 1:**
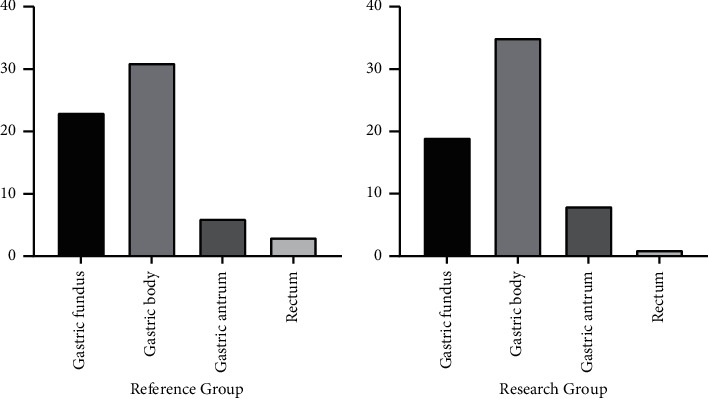
Comparison of lesion locations between the two groups (%).

Between November 2016 and May 2020, 126 patients assessed for eligibility and diagnosed with GIST in our institution were recruited. They were randomly assigned at a ratio of 1 : 1 to either the reference group (*n* = 63) or the research group (*n* = 63). Baseline characteristics of the patients of the research group (mean age of 52.67 ± 5.41 years, tumor size of 2.93–5.85 [4.17 ± 0.59] cm, lesion location: fundus in 23 cases, gastric body in 31 cases, gastric sinus in 6 cases, and rectum in 3 cases) were comparable with those of the reference group (mean age of 49.63 ± 5.52 years, tumor size of 3.48–4.89 (4.21 ± 0.34) cm, lesion location: fundus in 19 cases, gastric body in 35 cases, gastric sinus in 8 cases, and rectum in 1 case) (*P* > 0.05) (Table 1 and Figure 1).

### 2.2. Inclusion and Exclusion Criteria

Inclusion criteria: all eligible patients were diagnosed with GIST after examination; without recent use of other drugs; and provided written informed consent. This study was approved by the ethics committee of our hospital.

Exclusion criteria: patients with cardiovascular and cerebrovascular complications; with contraindications related to surgery; and with tumor metastases.

### 2.3. Methods

After preoperative examinations, patients in the reference group were given laparoscopic gastrectomy. Patients in the study group receive endoscopy-assisted laparoscopic gastrectomy: with the patient in a supine position, endotracheal intubation with intravenous compound general anesthesia was performed, followed by trocar puncture and pneumoperitoneum establishment with carbon dioxide, with the pneumoperitoneum pressure maintained at 12∼15 mmHg. The endoscope was properly placed, and the patient's lesion was accurately localized again by endoscopy-assisted laparoscopy according to the patient's preoperative examinations, followed by laparoscopic gastrointestinal tumor resection under the guidance of endoscopy. After surgery, specimens of the patient's lesions were collected to obtain the pathological diagnosis results. The patient was given basic postoperative treatment such as gastrointestinal decompression, nutritional support, and anti-infection.

### 2.4. Outcome

Before and after surgery, 1.8 ml venous blood was collected from the eligible patients for coagulation indexes determination, including prothrombin time (PT), activated partial thromboplastin time (APTT), thromboplastin time (TT), and fibrinogen (FIB). Intraoperatively, the operative time, surgical incision, and intraoperative bleeding were recorded for both groups to compare and analyze the patients' intraoperative conditions. The length of hospital stay, time to the first postoperative exhaustion, and duration of drainage tube and gastric tube dwelling of the two groups were recorded and compared. The levels of prealbumin (PA), transferrin (TRF), and albumin (ALB) in the two groups were recorded and monitored 3 days after surgery to assess the nutritional status of the two groups of patients. Complications including postoperative gastric fistula, abdominal infection, incisional infection, and gastrointestinal dysfunction were monitored and recorded to calculate the incidence of complications for the two groups.

### 2.5. Statistical Analysis

GraphPad Prism 8 software was used for image rendering, and SPSS22.0 software was used for data analyses. The count data were expressed as [n(%)] and subject to chi-square test, and the measurement data were represented by ($\bar{x}$ ± *s*) and processed by the *t*-test. Differences were considered statistically significant at *P* < 0.05.

## 3. Results

### 3.1. Coagulation Function

The two groups had similar preoperative coagulation levels of PT, APTT, TT, and FIB (*P* > 0.05). After surgery, there was no significant difference in APTT and TT levels between the two groups (*P* > 0.05). The research group showed lower PT levels (10.48 ± 0.68 vs. 11.97 ± 0.46) and higher FIB levels (0.67 ± 0.11 vs. 0.29 ± 0.07) versus those of the reference group (*P* < 0.05) (Tables 2 and 3).

### 3.2. Intraoperative Condition

The operative time, surgical incision, and intraoperative bleeding of patients in the research group were 81.21 ± 10.24 min, 3.63 ± 1.12 cm, and 18.74 ± 6.98 ml, respectively. Those of the reference group were 98.98 ± 15.31 min, 5.01 ± 1.14 cm, and 58.69 ± 15.87 ml. Endoscopy-assisted laparoscopic gastrectomy achieved better intraoperative conditions of patients versus laparoscopy alone (*P* < 0.05). (Table 4).

### 3.3. Postoperative Conditions

A markedly shorter length of hospital stay, time to the first postoperative exhaustion, and duration of drainage tube and gastric tube dwelling were observed in the research group (7.05 ± 1.31, 2.73 ± 1.02, 3.06 ± 0.67, and 1.62 ± 0.52) than in the reference group (10.56 ± 1.97, 5.33 ± 2.04, 5.87 ± 1.02, and 3.48 ± 0.96) (*P* < 0.05). (Table 5).

### 3.4. Nutrition Status

The PA, TRF, and ALB levels of patients in the research group at 3 days postoperatively (0.07 ± 0.02, 0.51 ± 0.11, and 7.03 ± 1.24) were higher than those in the reference group (0.12 ± 0.03, 0.68 ± 0.13, and 7.92 ± 1.02). Patients in the research group obtained better nutritional conditions versus those in the reference group (*P* < 0.05). (Table 6).

### 3.5. Complication

**Figure 2:**
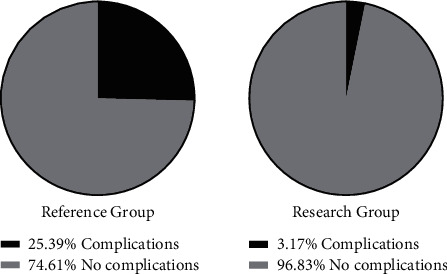
Incidence of complications in the two groups of patients (%).

The research group had 1 case of incisional infection and 1 case of gastrointestinal dysfunction, with an incidence of complications of 3.17%. The reference group had 3 cases of gastric fistula, 1 case of abdominal infection, 5 cases of incisional infection, and 7 cases of gastrointestinal dysfunction, with an incidence of complications of 25.39% (Table 7). The research group showed a significantly lower overall incidence of complications (2 (3.17%)) than the reference group (16 (25.39%)) (*x*^2^ = 12.704, *P* < 0.001) (Figure 2).

## 4. Discussion

Gastrointestinal stromal tumor (GIST) is a tumor of the mesenchymal tissue of the gastrointestinal tract with a high incidence of about 0.1–0.2 per 10,000 people [10]. The disease may be asymptomatic, and potential symptoms include abdominal pain, distension, vomiting blood, black stools, and abdominal masses [11]; however, the absence of specific symptoms and the insidiousness of the lesion complicate the effective diagnosis of the disease in the early stage [12]. The deficiency in the treatment efficiency of GIST is attributable to the small size and special location of tumors despite laparoscopic surgery as the choice of treatment with curable intent [13]. Moreover, the unsatisfactory surgical field of view of laparoscopy compromises the accurate assessment of surgical complications [14, 15]. The endoscopy-assisted laparoscopic gastrectomy is a new minimally invasive treatment protocol for GIST patients which allows the integration of endoscopy and laparoscopy to enhance the treatment efficiency [16]. Research has shown that endoscopy-assisted laparoscopic surgery provides effective recovery for GIST patients without serious complications [17, 18]. It has also been reported that such surgical protocol could minimize surgical trauma to ensure higher safety [19, 20]. Results of the present study showed superior coagulation indexes in the eligible patients given endoscopy-assisted laparoscopic gastrectomy versus laparoscopic gastrectomy alone, which indicates the benefits of the combined method in the protection and enhancement of coagulation functions. Besides, the combined protocol provides better intraoperative conditions for the patients, which may be attributed to the precision of tumor location and a small incision to abate intraoperative bleeding. Furthermore, patients in the research group obtained better improvements in postoperative conditions and nutritional status, indicating that endoscopy-assisted laparoscopy facilitates postoperative recovery and mitigates the negative impact of the surgery, which, consequently, results in a lower risk of complications, as evidenced by the lower incidence observed in the research group versus the reference group herein.

To sum up, endoscopy-assisted laparoscopic treatment shows significant improvements in the efficiency of minimally invasive surgery and ensures a better prognosis and quality of life of patients with a good safety profile, so it is worthy of clinical application.

## Figures and Tables

**Table 1:** Baseline characteristics of the two groups of patients ($\bar{x}$ ± *s*).

Groups	*n*	Gender	Mean age	Tumor size (cm)
Reference group	63	33/30	49.63 ± 5.52	4.21 ± 0.34
Research group	63	28/35	52.67 ± 5.41	4.17 ± 0.59
*t*	—	—	1.059	0.587
*P*	—	—	0.292	0.558

**Table 2:** Comparison of preoperative coagulation indexes between the two groups of patients ($\bar{x}$ ± *s*).

Groups	*n*	Before surgery			
		PT (s)	APTT (s)	TT (s)	FIB (g/L)
Reference group	63	12.41 ± 1.54	29.65 ± 3.71	13.01 ± 1.21	0.11 ± 0.05
Research group	63	12.48 ± 1.49	29.41 ± 3.89	13.42 ± 1.13	0.12 ± 0.03
*t*	—	0.223	0.354	1.973	1.270
*P*	—	0.824	0.724	0.051	0.206

**Table 3:** Comparison of postoperative coagulation indexes between the two groups of patients ($\bar{x}$ ± *s*).

Groups	*n*	After surgery			
		PT (s)	APTT (s)	TT (s)	FIB (g/L)
Reference group	63	11.97 ± 0.46	28.42 ± 0.78	12.74 ± 1.05	0.29 ± 0.07
Research group	63	10.48 ± 0.68	28.35 ± 1.01	12.46 ± 1.07	0.67 ± 0.11
*t*	—	14.428	0.497	1.535	25.014
*P*	—	<0.001	0.620	0.127	<0.001

**Table 4:** Comparison of intraoperative conditions between the two groups of patients ($\bar{x}$ ± *s*).

Groups	*n*	Operative time (min)	Surgical incision (cm)	Intraoperative bleeding (ml)
Reference group	63	98.98 ± 15.31	5.01 ± 1.14	58.69 ± 15.87
Research group	63	81.21 ± 10.24	3.63 ± 1.12	18.74 ± 6.98
*t*	—	7.653	6.854	18.287
*P*	—	<0.001	<0.001	<0.001

**Table 5:** Comparison of postoperative conditions between the two groups of patients ($\bar{x}$ ± *s*, *d*).

Groups	*n*	Length of hospital stay	Time to the first postoperative exhaustion	Duration of drainage tube dwelling	Duration of gastric tube dwelling
Reference group	63	10.56 ± 1.97	5.33 ± 2.04	5.87 ± 1.02	3.48 ± 0.96
Research group	63	7.05 ± 1.31	2.73 ± 1.02	3.06 ± 0.67	1.62 ± 0.52
*t*	—	11.745	9.061	18.234	13.441
*P*	—	<0.001	<0.001	<0.001	<0.001

**Table 6:** Comparison of the 3-d postoperative nutritional status of patients in the two groups ($\bar{x}$ ± *s*).

Groups	*n*	PA (ng/L)	TRF (ng/L)	ALB (g/L)
Reference group	63	0.07 ± 0.02	0.51 ± 0.11	7.03 ± 1.24
Research group	63	0.12 ± 0.03	0.68 ± 0.13	7.92 ± 1.02
*t*	—	11.230	7.746	4.398
*P*	—	<0.001	<0.001	<0.001

**Table 7:** Comparison of the complications between the two groups of patients (%).

Groups	*n*	Gastric fistula	Abdominal infection	Incisional infection	Gastrointestinal dysfunction
Reference group	63	3 (4.76)	1 (1.59)	5 (7.94)	7 (11.11)
Research group	63	0 (0.00)	0 (0.00)	1 (1.59)	1 (1.59)
*x* ^2^			12.704		
*P*			<0.001		

## Data Availability

The datasets used during the present study are available from the corresponding author upon reasonable request.

## References

[1] Akahoshi K., Oya M., Koga T., Shiratsuchi Y. (2018). Current clinical management of gastrointestinal stromal tumor. *World Journal of Gastroenterology*.

[2] Ahmed M. (2020). Recent advances in the management of gastrointestinal stromal tumor. *World Journal of Clinical Cases*.

[3] Lee W.-J., Chan C.-P., Wang B.-Y. (2013). Recent advances in laparoscopic surgery. *Asian Journal of Endoscopic Surgery*.

[4] Hobeika C., Sabbagh C., Najah H., Eveno C. (2017). Laparoscopic exploration for peritoneal carcinomatosis: surgical technique. *Journal of Visceral Surgery*.

[5] Lee C. M., Park S. (2017). Laparoscopic techniques and strategies for gastrointestinal GISTs. *The Journal of Visualized Surgery*.

[6] Majinyang S., Ruth Y. K. M., Ahmed S. (2019). Microscopically positive resection margins in laparoscopic gastric GIST resection may not confer a poorer prognosis. *Surgical Laparoscopy Endoscopy & Percutaneous Techniques*.

[7] Arora E. (2021). Laparoscopic transgastric resection of a large gastric GIST: a case report and review of literature. *Surgery Journal*.

[8] Rodriguez J. H., Ponsky J. L. (2020). Operating with the endoscope. *Surgical Clinics of North America*.

[9] Mantese G. (2019). Gastrointestinal stromal tumor. *Current Opinion in Gastroenterology*.

[10] El-Menyar A., Mekkodathil A., Al-Thani H. (2017). Diagnosis and management of gastrointestinal stromal tumors: an up-to-date literature review. *Journal of Cancer Research and Therapeutics*.

[11] Rajravelu R. K., Ginsberg G. G. (2020). Management of gastric GI stromal tumors: getting the GIST of it. *Gastrointestinal Endoscopy*.

[12] Chernousov A. F., Vetshev F. P., Vychuzhanin D. V. (2020). Laparoscopic and robot-assisted procedures in patients with gastrointestinal stromal tumors (GIST) of stomach. *Khirurgiya. Zhurnal im. N.I. Pirogova*.

[13] Vitiello G., Ye Zhou J. H., Fernandez-Ananin S., Balague-Ponz C. (2021). Laparoscopic resection of duodenal GIST. *Cirugía Española*.

[14] Loureiro M. d. P., Almeida R. A. A. d., Claus C. M. P. (2016). Laparoscopic resection OF gastrointestinal stromal tumors (GIST). *ABCD. Arquivos Brasileiros de Cirurgia Digestiva (São Paulo)*.

[15] Florin C. M., Bogdan F, Cristian L, Maria T. A, Mihai D, Viorel S (2020). Surgical treatment of gastric GIST: feasibility of laparoscopic resection and postoperative outcome. *Journal of the College of Physicians and Surgeons--Pakistan: JCPSP*.

[16] Tantia M. (2021). Gastric glomus tumour: a case report. *Journal of Minimal Access Surgery*.

[17] Mazer L., Worth P., Visser B. (2021). Minimally invasive options for gastrointestinal stromal tumors of the stomach. *Surgical Endoscopy*.

[18] Hagerty B. L., Torres M. B., Drake J. (2021). Trends and predictors of failure of minimally invasive surgery for gastric GIST. *Journal of Gastrointestinal Surgery*.

[19] Sista F., De Leonardis M., Carandina S., Pessia B., Clementi M., Vicentini R. (2021). Surgical management of rectal GIST. A case report and a review of literature. *Annali Italiani di Chirurgia*.

[20] Yu M., Wang D.-C., Wei J., Lei Y.-H., Fu Z.-J., Yang Y.-H. (2021). Meta-analysis on the efficacy and safety of laparoscopic surgery for large gastric gastrointestinal stromal tumors. *The American Surgeon*.

